# The role of FOSL1 in stem-like cell reprogramming processes

**DOI:** 10.1038/s41598-021-94072-0

**Published:** 2021-07-19

**Authors:** Valeria Pecce, Antonella Verrienti, Giulia Fiscon, Marialuisa Sponziello, Federica Conte, Luana Abballe, Cosimo Durante, Lorenzo Farina, Sebastiano Filetti, Paola Paci

**Affiliations:** 1 Department of Translational and Precision Medicine, Sapienza University of Rome, 00161 Rome, Italy; 2 Fondazione Per La Medicina Personalizzata, Via Goffredo Mameli, 3/1, Genoa, Italy; 3 Institute for Systems Analysis and Computer Science “Antonio Ruberti”, National Research Council, Rome, Italy; 4 Department of Pediatric Hematology/Oncology and Cell and Gene Therapy, IRCCS, Ospedale Pediatrico Bambino Gesù, Piazza Sant’Onofrio 4, 00165 Rome, Italy; 5 Department of Computer, Control, and Management Engineering, Sapienza University of Rome, Rome, Italy; 6 School of Health, Unitelma Sapienza University of Rome, Rome, Italy

**Keywords:** Cell biology, Cancer

## Abstract

Cancer stem-like cells (CSCs) have self-renewal abilities responsible for cancer progression, therapy resistance, and metastatic growth. The glioblastoma stem-like cells are the most studied among CSC populations. A recent study identified four transcription factors (SOX2, SALL2, OLIG2, and POU3F2) as the minimal core sufficient to reprogram differentiated glioblastoma (GBM) cells into stem-like cells. Transcriptomic data of GBM tissues and cell lines from two different datasets were then analyzed by the SWItch Miner (SWIM), a network-based software, and FOSL1 was identified as a putative regulator of the previously identified minimal core. Herein, we selected NTERA-2 and HEK293T cells to perform an in vitro study to investigate the role of FOSL1 in the reprogramming mechanisms. We transfected the two cell lines with a constitutive FOSL1 cDNA plasmid. We demonstrated that FOSL1 directly regulates the four transcription factors binding their promoter regions, is involved in the deregulation of several stemness markers, and reduces the cells’ ability to generate aggregates increasing the extracellular matrix component FN1. Although further experiments are necessary, our data suggest that FOSL1 reprograms the stemness by regulating the core of the four transcription factors.

## Introduction

Cancer stem-like cells are cancer cells that share many of the same defining characteristics with stem cells, including self-renewal (i.e. division with maintenance of the undifferentiated state), the capability to develop into multiple lineages, and the potential to proliferate extensively^1^. The regulation of stem cell fate promotes cancer initiation, progression, and metastatic growth^2^. Although stem-like cells represent a small subpopulation in the tumor mass, they can be more quiescent and resistant to toxins and chemicals and contribute to cancer therapy resistance^2^.

Cancer stem cells were first evidenced in acute myeloid leukemia^3^ and subsequently in other tumors, including glioblastoma multiforme (GBM)^4,5^. In particular, several studies on GBM identified the presence of a stem-like cell subpopulation with radiotherapy- and chemotherapy-resistant properties that contributed to tumor initiation, progression, treatment resistance, and relapse^6^. Due to their ability to self-renew, proliferate, and differentiate into multiple lineages, these cells were termed glioblastoma stem-like cells (GSCs) and several studies have been conducted in order to better characterize them. It has been reported that they express cell surface markers such as CD133^7^, SSEA-1^8^, CD44^9^, and integrin a 6^10^. GSCs are believed to play a large role in carcinogenesis^11,12^. Since GSCs are a major reason for the ineffectiveness of current therapies for glioblastoma and other cancers., the study of cancer stem-like cell features may pave the way for the development of novel therapeutic strategies. One study published in the last years experimentally demonstrated that induction of the expression of four neurodevelopmental transcription factors (TFs) was sufficient to reprogram differentiated glioblastoma cells into stem-like cells^13^. These four TFs are: (1) *SOX2*, an intron-less gene that encodes a member of the SOX (SRY-related HMG box) family of TFs involved in the regulation of embryonic development and in the determination of cell fate, which is required for stem cell maintenance and gene expression regulation^14^; (2) *OLIG2*, a gene that encodes a basic helix-loop-helix TF expressed in oligodendroglial tumors of the brain and whose protein is a regulator of neuroectodermal progenitor cell fate^15,16^; (3) *POU3F2*, a gene that encodes a member of the POU-III class of neural TFs and is involved in neuronal differentiation^17,18^; and (4) *SALL2*, which is widely expressed in the brain and may have a role in promoting neuronal and eye development^19^.

Identification of the upstream molecular mechanisms that regulate the four TFs is a critical goal, with broad implications in terms of diagnosis and therapy. A recent network-based study^20^ identified only one regulator of the four TFs when applying SWItch Miner (SWIM) software on two datasets of gene expression data from human glioblastoma tissues and cell lines^21^. Using SWIM to search for switch genes shared by both analyzed glioblastoma datasets, the authors identified FOS-like transcription factor (*FOSL1*) as the most promising one.

Several findings suggest a possible role of FOSL1 in stemness. Indeed, *FOSL1* was downregulated in stem-like cells and negatively correlated with the four TFs identified by Suvà and colleagues^13,21^. It is well known that CSC phenotype and epithelial-to-mesenchymal transition (EMT) are intimately interconnected^22^. Interestingly, FOSL1 was positively correlated with genes encoding proteins crucial for cell matrix adhesion (e.g. FN1 and a-actin), a key process involved in the EMT^21^. Therefore, FOSL1 expression could promote cell differentiation by repressing the four TFs and the EMT. However, few experimental data exist on the direct involvement of FOSL1 in stemness mechanisms.

In this paper we validated by in vitro experiments the role of FOSL1 in regulating the four neurodevelopmental TFs involved in the differentiation process of GSCs, as previously suggested by our computational study^21^. Moreover, we provided evidence on the effect of FOSL1 on the expression of several stemness markers and on the modulation of cell–matrix adhesion.

## Results

### FOSL1 negatively regulates four stem-like associated TFs in NTERA-2 and HEK293T cell lines

Given the role of the four TFs (*SALL2*, *SOX2*, *OLIG2*, and *POU3F2*) in reprogramming differentiated glioblastoma cells into stem-like cells^13^ and the potential role of *FOSL1* in regulating them as postulated by Fiscon and colleagues, our first goal was to investigate the molecular relationship between *FOSL1* and the four TFs.

**Figure 1:**
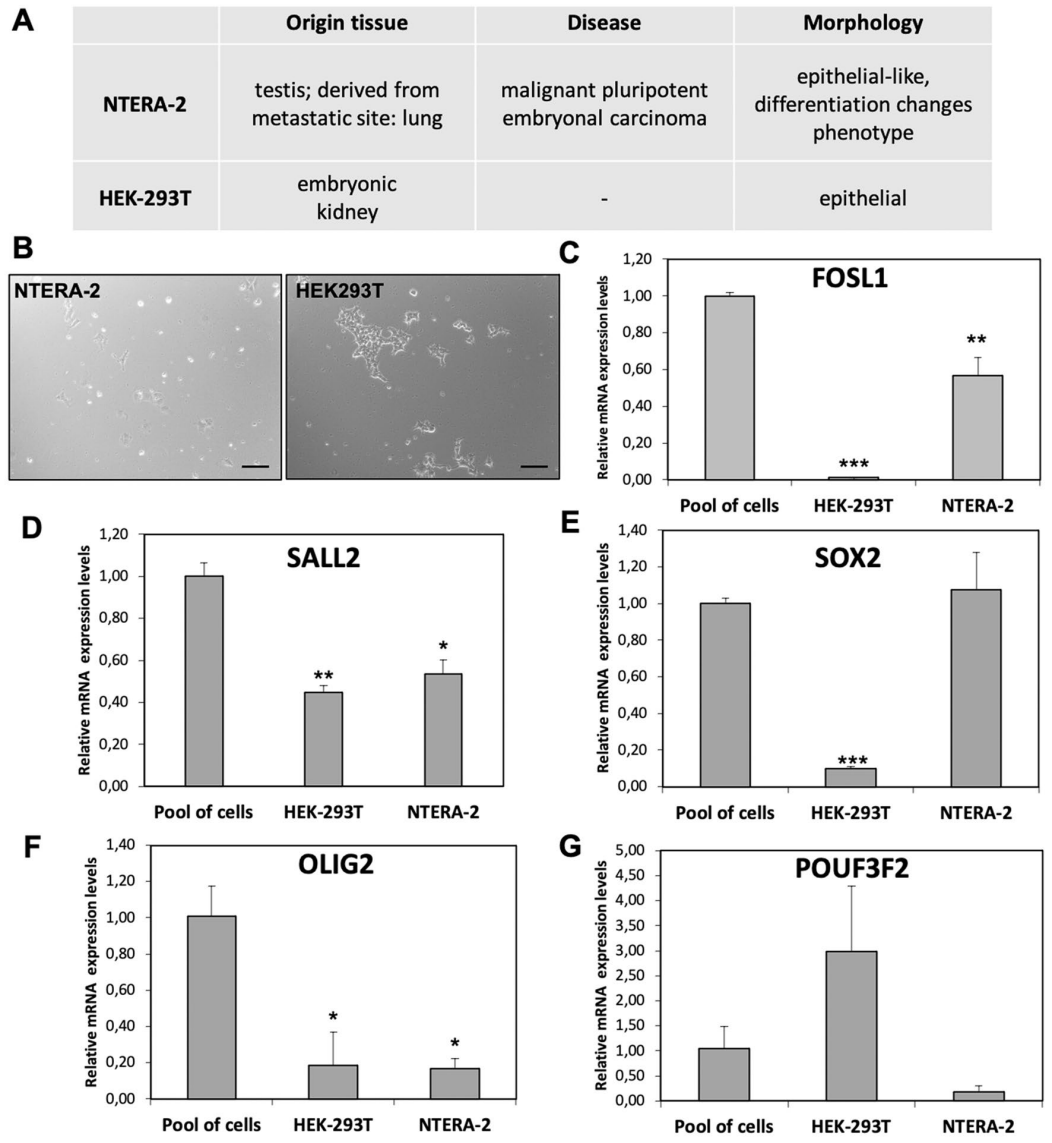
Cell lines characteristics and TFs expression levels. (**A**) Summary of cell lines characteristics. (**B**) Morphology of the NTERA-2 and HEK293T, 10X magnification, bar length 200 µm. (**C**–**G**) Relative expression levels of FOSL1, SALL2, SOX2, OLIG2 and PUO3F2. The Pool of cells sample is a pool of equimolar cDNA of 8 commercial cell lines used as control sample (HEK293T, NTERA-2, HeLa, NthyORI, 8505c, SW1736, TT, MZ-CRC1). Data are presented as mean ± standard deviation of the relative expression of each target, corrected for the *GAPDH* expression (housekeeping gene), and compared with the expression level of the same target in the pool of cells (calibrator). *p* value < 0.05, *; 0.005 **; 0.0005 *** (t-test data).

We used the expression data reported in Human Protein Atlas database^23^ to select two cell lines suitable for this purpose, the NTERA-2 and HEK293T cell lines, both with low *FOSL1* expression levels and discrete levels of the other four TFs. Moreover, the NTERA-2 and HEK293T are embryonic arising cell lines. In particular, NTERA-2 line arises from a malignant pluripotent embryonal carcinoma, and the HEK293T line arises from an embryonic kidney. A recent study demonstrated HEK293T stemness features^24^. The origin and characteristics of both cell lines are summarized in Fig. 1A.

As represented in Fig. 1, we verified the expression levels of all TFs in both cell lines. In order to obtain an overview of the relative expression levels of all TFs, we compared the results in NTERA-2 and HEK293T using a pool of equimolar cDNA of 8 immortalized cell lines as control sample.

The HEK293T cell line showed high levels of *POU3F2*, moderate levels of *SALL2*, and low levels of *FOSL1*, *SOX2*, and *OLIG2*. The NTERA-2 cell line showed high levels of *SOX2*, moderate levels of *SALL2* and *FOSL1*, and low levels of *OLIG2* and *POU3F2*.

Since we confirmed low *FOSL1* expression levels in both cell lines, as reported in the Human Protein Atlas, we transfected HEK293T and NTERA-2 cell lines with a construct containing *FOSL1* cDNA (pCMV-FOSL1). Using real-time polymerase chain reaction (PCR), we analyzed the expression levels of the four TFs after 48 h from transfection and compared the levels with those obtained in cell lines transfected with the control vector (empty vector).

**Figure 2:**
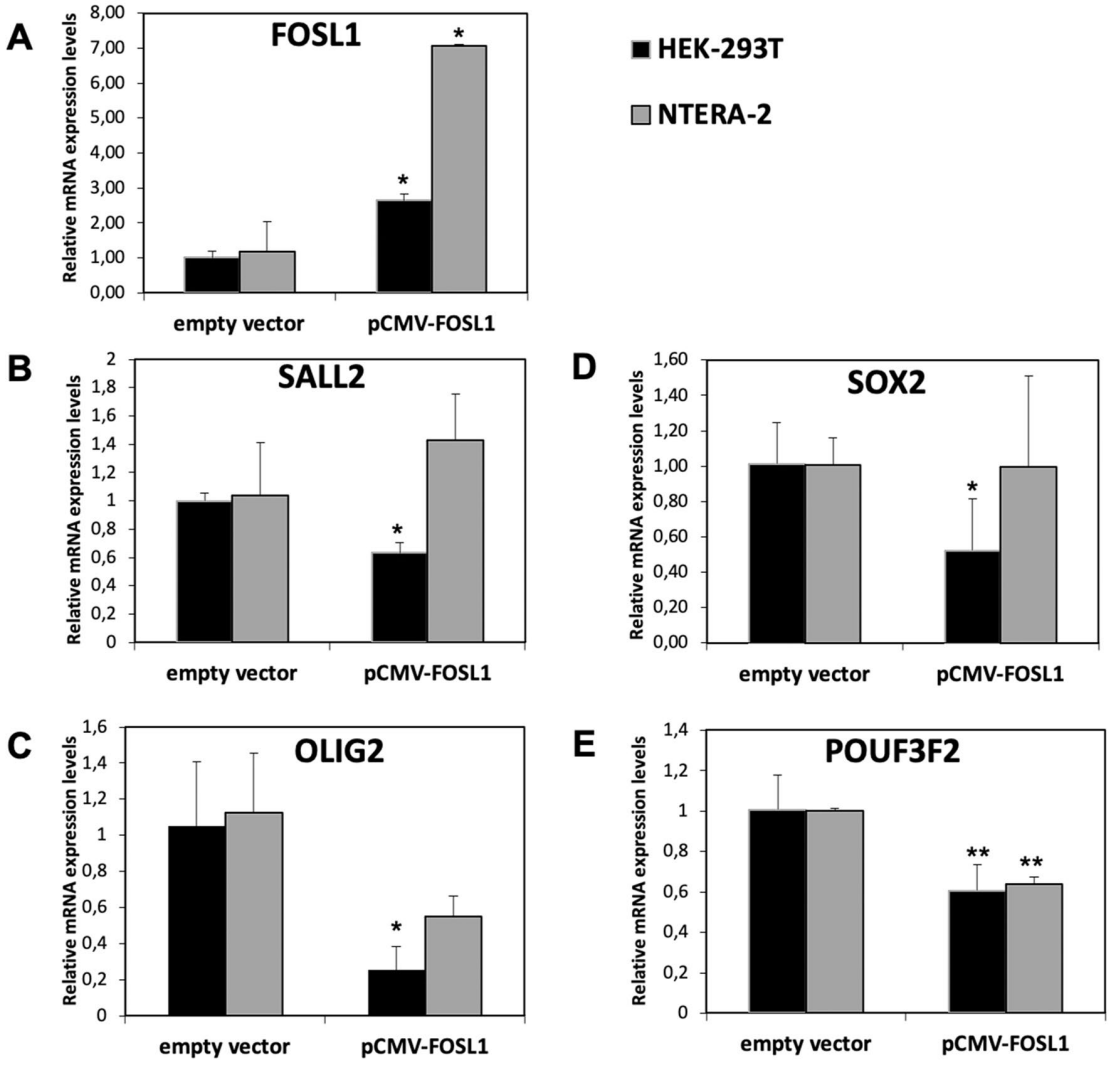
Gene expression analysis of *FOSL1* in overexpressed cells. Relative expression levels of *FOSL1* (**A**), *SALL2* (**B**), *OLIG2* (**C**), *SOX2* (**D**), and *PUO3F2* (**E**) in NTERA-2 and HEK293T cells lines after 48 h from transfection with the empty vector or pCMV-FOSL1 vector. Data are presented as mean and standard deviation of the relative expression of each target using the *GAPDH* as housekeeping gene and the empty vector as calibrator. *p* value < 0.05, *; 0.005 **; 0.0005 *** (t-test data).

Gene expression analysis performed before and after transfection revealed reduced expression of the four TFs in the HEK293T cell line and reduced expression of *OLIG2* and *POU3F2* in the NTERA-2 cell line (Fig. 2), suggesting that the switch gene *FOSL1* negatively regulates the four TFs.

### FOSL1 directly interacts with four TFs promoters

In light of previous results, we used the HEK293T cell line to investigate the molecular mechanisms behind the expression level changes of the four TFs after *FOSL1* overexpression. In order to determine whether FOSL1 directly or indirectly regulates expression level changes of the four TFs, we performed a chromatin immunoprecipitation (ChIP) experiment to verify whether FOSL1 binds the promoter regions of the four TFs. We selected the promoter of the four TFs considering the genomic regions upstream of the start of transcription containing the *FOSL1* consensus binding sequence as previously described in^20^ and reported in the Jaspar database (http://jaspar.genereg.net/matrix/MA0477.2/).

As shown in Supplementary Fig. 1, we extracted and sonicated chromatin from HEK293T cell lines transfected with the pCMV empty vector or containing *FOSL1* (pCMV-FOSL1). Immunoprecipitation was performed using a specific antibody for FOSL1.

**Figure 3:**
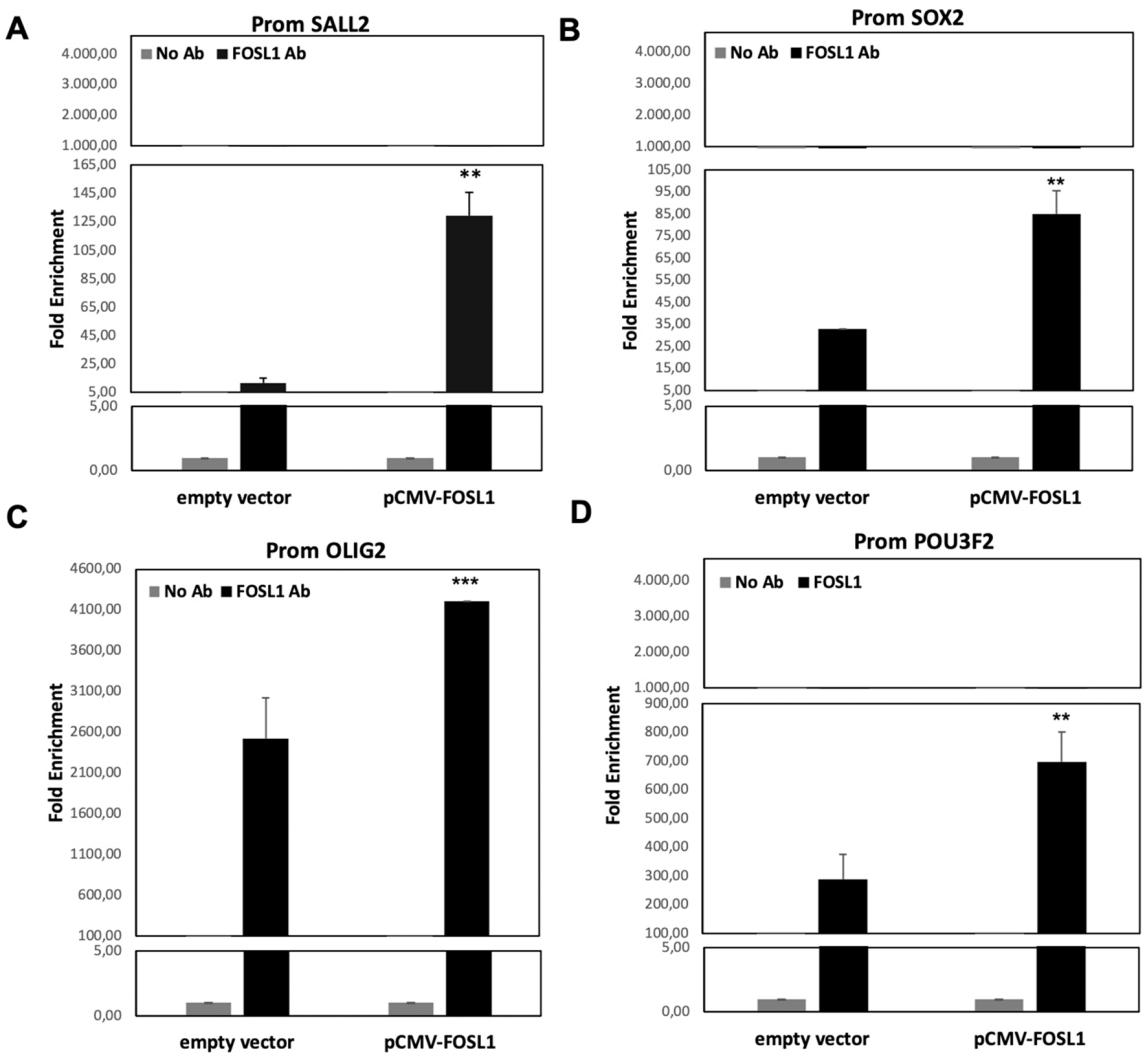
ChIP-Real Time with FOSL1 antibody. Real-time PCR results obtained by ChIp experiment performed on HEK293T cells after 48H from transfection with pCMV-FOSL1 vector. Histograms represent the quantification levels of the promoter regions of (**A**) SALL2, (**B**) SOX2, (**C**) OLIG2, (**D**) POU3F2. The results are normalized for input sample (the chromatin extracted from treated cells) and expressed as fold enrichment of the promoter region of the four TFs found in the FOSL1 Ab samples. *p* value < 0.05, *; 0.005 **; 0.0005 *** (t-test data).

Using specific primers**,** we compared the quantity of the selected promoter regions, for the four TFs, in samples immunoprecipitated with FOSL1 antibody and control samples (No AB). Using real-time PCR, we found an enrichment relative to promoter regions of all four TFs in the immunoprecipitated samples if compared with controls. As shown in Fig. 3, we found an enrichment of 150-fold for *SALL2* and *SOX2* promoter regions, 900-fold for *POU3F2* promoter region and 4000-fold for *OLIG2* promoter region.

Taken together, these results suggest that FOSL1 directly regulates the four TFs, binding their promoter regions.

### FOSL1 effects on staminal markers

Since we demonstrated that FOSL1 directly regulates the expression of the four TFs involved in GBM stemness, we investigated the effect of FOSL1 in the stemness process. We analyzed the expression levels of 15 pluripotent stem cell markers in the total protein extract from HEK293T cells transfected with empty vector or pCMV-FOSL1 vector.

**Figure 4:**
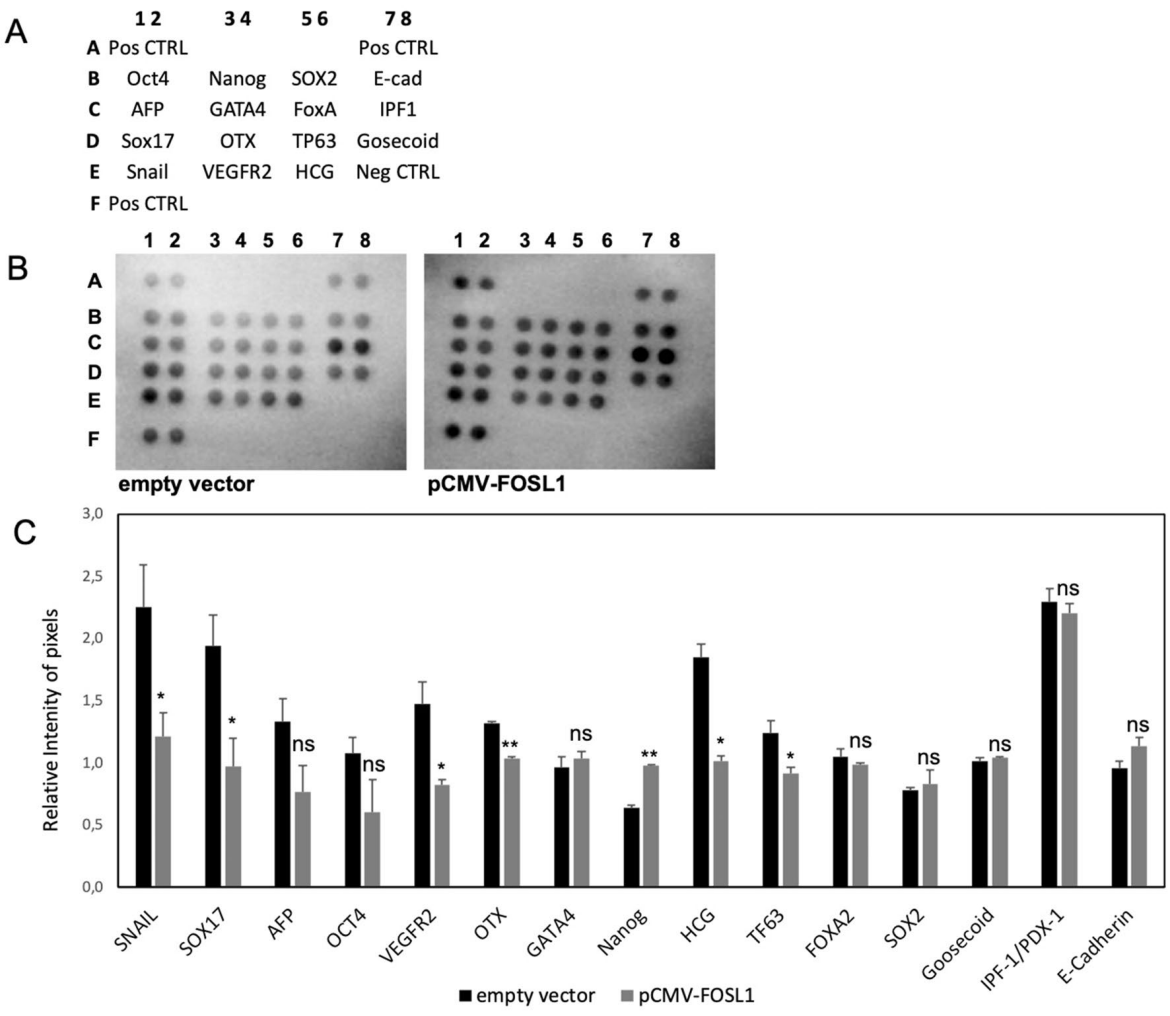
Staminal markers analysis after the overexpression of FOSL1. (**A**) Reference positions of the antibodies spotted on the proteome array. (**B**) Image of the acquisition of the two membranes hybridized with the total protein extract from HEK293T cells transfected for 48 h with empty vector or pCMV-FOSL1. (**C**) Graphical representation of the pixel quantification of each spot. Data were represented as the mean ± standard deviation of the technical duplicates of the spots presented on the arrays. Results have been normalized for the mean of the positive control spots (3 couple in each membrane). *p* value < 0.05, *; 0.005 **; 0.0005 *** (t-test data).

As shown in Fig. 4, after the overexpression of FOSL1, we observed a statistically significant deregulation of 7 of the 15 stemness markers analyzed. In particular, 6 markers were down regulated (SNAIL, SOX17, VEGFR2, OTX, HCG, and TP63) and one was upregulated (NANOG).

These results highlight the role of FOSL1 in stemness processes.

### FOSL1 reduces the cells’ ability to generate aggregates increasing the extracellular matrix component FN1

According to results obtained by Fiscon et al.^21^, *FOSL1* seems to directly correlate with higher levels of extracellular matrix and focal adhesion components. In order to investigate the relationship between FOSL1 and extracellular components, we analyzed the ability of cells to generate aggregates in the extracellular matrix mix from Engelbreth-Holm-Swarm cells, the Matrigel. HEK293T cell lines transfected with *FOSL1* (pCMW-FOSL1) or the empty vector were photographed every two days. After one week, the cells formed aggregates.

**Figure 5:**
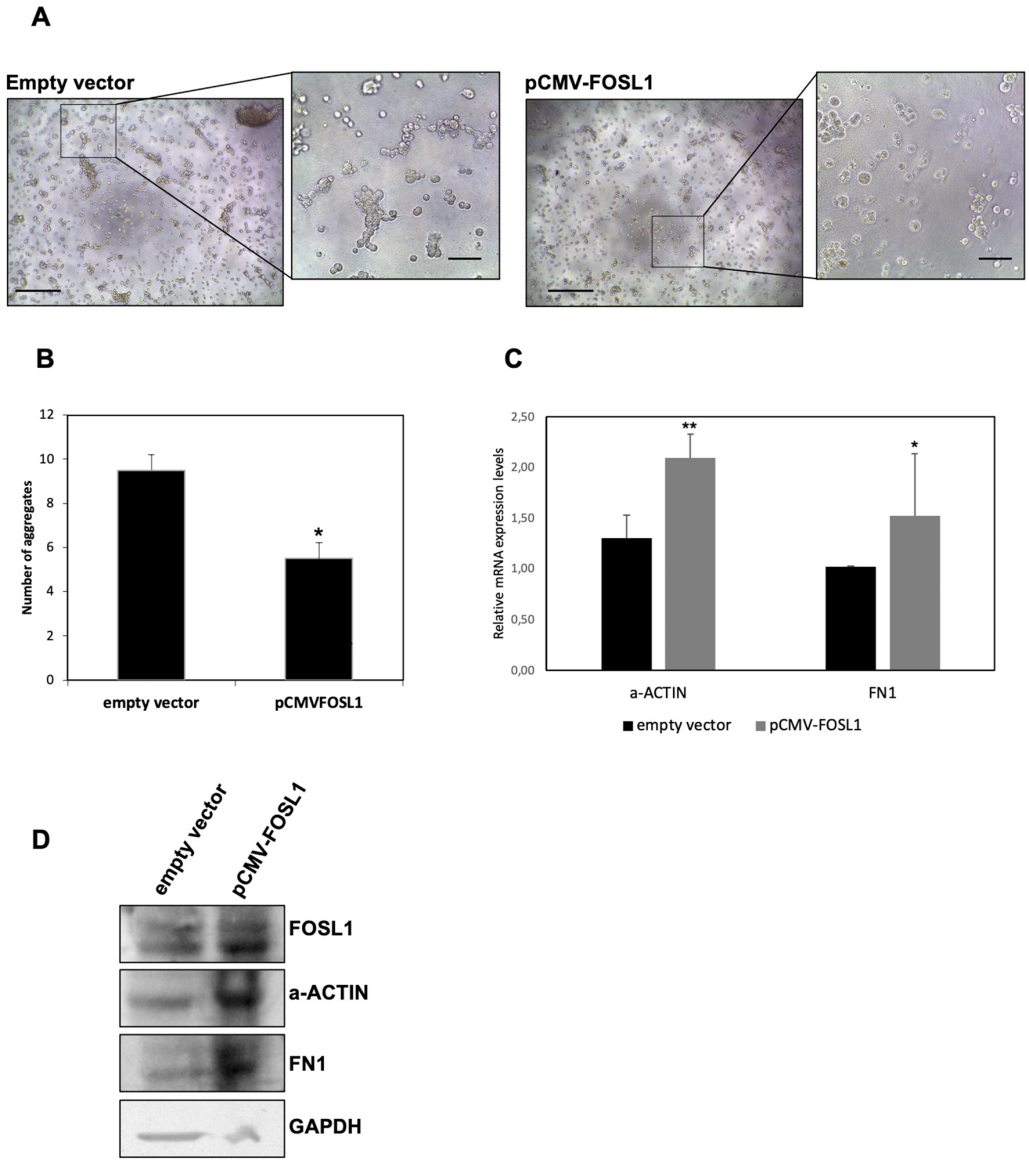
Cell aggregation assay. HEK293T after 1 week from the transfection with empty vector or pCMV-FOSL1 vector. (**A**) Photographs were performed at 5X magnification (scale bar length: 500 μm) and the particular were acquired at 20X magnification (scale bar length: 200 μm). (**B**) Number of aggregates in a field of view at 20X magnification (**C**) Expression analysis of a-ACTIN and *FN1* in HEK293T cells after 48 h from transfection with pCMV-FOSL1 plasmid. Data are presented as mean ± standard deviation of the relative expression of each target using the *GAPDH* expression levels as endogenous control and the empty vector as calibrator. *p* value < 0.05, *; 0.005 **; 0.0005 *** (t-test data). (**D**) Protein levels in HEK293T cells after 48 h from transfection with empty vector or pCMV-FOSL1 plasmid, in the figure were reported the representative western blot of detection of FOSL1, FN1, a-ACTIN and GAPDH (used as loading control).

As shown in Fig. 5A, cells that overexpressed *FOSL1* seemed to generate less aggregates than cells transfected with the empty vector. This suggests a lower motility of cells transfected with *FOSL1*, which may be due to the greater quantity of extracellular matrix components expressed in these cells when *FOSL1* was overexpressed. The number of aggregates formed by each cell lines in the different conditions analyzed are reported in Fig. 5B.

We confirmed data analyzing the levels of expression of a-ACTIN, one of the cytoskeleton marker that influence cell behavior interacting with the extracellular matrix^25^, and an extracellular matrix component, FN1. As shown in Fig. 5C, in HEK293T cell lines after 48 h from transfection with pCMV-FOSL1, we found statistically significant higher expression levels of FN1 and a-ACTIN. Using western blot, we confirmed the FOSL1 overexpression and the higher levels of a-ACTIN and FN1, Fig. 5D. Entire membranes are reported in Supplementary Fig. 2.

## Discussion

In recent decades, much attention has been paid to cancer stem-like cells, which represent the critical subset within the tumor mass responsible for perpetuating the tumor, leading to its aggressiveness and contributing to therapy resistance, recurrence, and metastasis. Several efforts have been made to discover novel therapeutic strategies that could promote the differentiation of cancer stem-like cells in order to halt cancer growth and potentially affect patient outcome.

Increasing evidence suggests that modulation of the expression of a small set of TFs is able to maintain the stem-like phenotype and prevent cell differentiation. In particular, Suvà and colleagues identified four neurodevelopmental TFs (i.e., *SOX2*, *SALL2*, *OLIG2*, and *PUO3F2*) that were selectively expressed in GSCs and whose induction was sufficient to fully reprogram differentiated cells into GSCs. Thus, the identification of an upstream pathway that regulates the four TFs could be crucial to control cancer stem-like cell differentiation in human glioblastoma and to serve as a successful target for therapeutic strategies. With this aim, we based our study on the results of Fiscon and colleagues^26^, who applied SWIM algorithms to two datasets of gene expression analysis from glioblastoma tissues and cell lines.

SWIM was developed to computationally identify key genes (denoted as “switch genes”) that are likely to be critically associated with drastic changes in cell or tissue phenotype. SWIM phenotype-specific applications are broad and include the identification of switch genes in organisms, complex diseases, and cancers^26–29^.

SWIM was able to identify important switch genes involved in the transition from a stem-like to a differentiated phenotype of glioblastoma cells. Among the switch genes common to both datasets, the authors found *FOSL1*, which was down-regulated in stem-like cells and highly negatively correlated with the four TFs. Moreover, the authors found a predicted FOSL1 consensus binding motif in the regulatory regions of all four TFs that showed a positive correlation with proteins crucial for cell matrix adhesion and cell motility, including actin, collagen, fibronectin, and several integrins^26^. Based on these findings, the authors identified *FOSL1* as a promising candidate to orchestrate the differentiation of cancer stem-like cells by repressing expression of the four TFs and restoring the physiological equilibrium between cell adhesion and migration, thus interfering with cancer progression.

FOSL1 is a well-known transcription factor. Earlier studies on FOSL1 demonstrated its involvement in embryonic development and bone formation. FOSL1 is abnormally expressed in many tumors and plays an important role in tumorigenesis and progression^30,31^. It is mainly regulated by the mitogen-activated protein kinase (MAPK) signaling pathway^30^, the most heavily involved pathway in cancer progression. Interestingly, the abnormal expression of FOSL1 in various tumors and its effects on tumor progression differ according to tumor type^30^.

The results presented in this paper experimentally validate the predicted role of *FOSL1* as a possible regulator of stemness. We first analyzed expression levels of the four TFs after FOSL1 overexpression in a pluripotent human embryonal carcinoma cell line (NTERA-2) and in a human embryonic kidney 293 cell line (HEK-293T) selected due to low FOSL1 expression levels and discrete levels of the other four TFs. After the FOSL1 overexpression, we observed a downregulation of all the four TFs in the HEK-293T cells and of POUF3F2 in NTERA-2 cells. The difference observed between the two cell lines may be due to the different origins of the cells and to the different response of each cell line to the treatment.

We then used ChIP in HEK-293T transfected cells to demonstrate that FOSL1 directly regulates *OLIG2, SALL2, SOX2* and *POU3F2* expression levels through the binding of their promoter regions. In order to better investigate the role of FOSL1 in the stemness processes, we analyzed the levels of 15 proteins with a commercial proteome array in FOSL1 overexpressed cells. Seven of the 15 well-known stemness markers analyzed resulted to be deregulated after the overexpression of FOSL1. All the deregulated markers are involved in the epithelial-mesenchymal transition (EMT) in cancer or confer a more aggressive phenotype to the cells^32,33^. Among them, we found a reduction of SOX17, an EMT-suppressor able to transcriptionally repress *FN1*, a gene that codifies for a protein involved in ECM remodeling during EMT^33,34^. Interestingly, in our previous computational study, we found that FOSL1 positively correlated with genes encoding proteins crucial for cell–matrix adhesion, including FN1 and a-actin. We confirmed that the overexpression of FOSL1 induced a modulation in the cell–matrix adhesion, as demonstrated by the increase in the cell aggregates observed in a semisolid condition and by the induction of FN1 and a-actin expression.

## Conclusions

Overall, our experimental results validate SWIM analysis predictions^21^, confirming the potential role of *FOSL1* as a crucial factor in modulating the expression levels of the four TFs, and consequently cancer stem-like conditions. This may suggest that novel therapeutic strategies able to restore *FOSL1* expression may be effective for glioblastoma treatment.

### Limitations of the study

Our study on the role of *FOSL1* in stemness was based on data obtained from gene expression analysis in glioblastoma. The most significant limitation of our study was that we could not find glioblastoma lines to confirm the data obtained with the commercial lines NTERA-2 and HEK293T.

## Materials and methods

### Cell line culture and transfection

NTERA-2 and HEK-293 T cell lines (provided by Dr. E. Ferretti) were cultured in Dulbecco’s Modified Eagle Medium (Gibco-BRL Division, Thermo Fisher Scientific, Waltham, Massachusetts, USA) containing 10% fetal bovine serum (Gibco-BRL Division, Thermo Fisher Scientific, Waltham, Massachusetts, USA) and antibiotic–antimycotic solution (Gibco-BRL Division, Thermo Fisher Scientific, Waltham, Massachusetts, USA) and incubated at 37 °C in an atmosphere of 5% CO2 as previously described in^35^.

Cell lines were transfected with pCMV6 empty vector or the complementary DNA (cDNA) of FOSL1 (NM_005438). The pCMV-FOSL1 was obtained from OriGene and the empty vector was obtained from a pCMV-FOSL1 clone digested with EcoRI and XhoI restriction enzymes. Cell lines were transfected with empty vector and pCMV-FOSL1 using Lipofectamine 3000 (Invitrogen Division, Thermo Fisher Scientific, Waltham, Massachusetts, USA) according to the manufacturer’s instructions and described in^36^.

Cells were plated the day before transfection at 80% of confluent in a 6-well-plate (80,000 cells/well) and starved for two hours before transfection. In each starved well was added OptiMEM medium (200 μl) containing lipofectamine 3000 and P3000 (5 μl each) and plasmids (2 μg).

Starvation was performed using Opti-MEM medium (Gibco-BRL Division, Thermo Fisher Scientific, Waltham, Massachusetts, USA) without supplements. Cells were harvested after 48 h from transfection.

### RNA isolation and gene expression analysis

The RNeasy Mini Kit (Qiagen, Hilden, Germany) was used to isolate total RNA from transfected cell cultures. RNA concentrations were measured with the NanoDrop 2000/2000c Spectrophotometers (Thermo Fisher Scientific, Waltham, Massachusetts, USA). First-strand cDNA was synthesized using the High Capacity cDNA Reverse Transcription kit (Thermo Fisher Scientific, Waltham, Massachusetts, USA).

Real-time PCR analyses were performed using a 7900HT Fast Real-Time PCR System, and SDS 2.3 software (both from Thermo Fisher Scientific, Waltham, Massachusetts, USA) was used to calculate cycle threshold values^37^. SYBR Green Master Mix was used to perform quantitative analyses with the specific primers reported in Table 1 using a standard protocol according to the manufacturer’s instructions (Thermo Fisher Scientific, Waltham, Massachusetts, USA). *GAPDH* was used as endogenous control. Final results were calculated using the 2 − ΔΔCt method and normalized to the calibrator sample.

**Table 1:** Primer sequences and application.

Primer name	Sequence	Application
FOSL1 F1	ACCTACCCTCAGTACAGCCC	Real time PCR
FOSL1 R1	TGCAGCCCAGATTTCTCATCT	
SOX2 F2	CAACCAGAAAAACAGCCCGG	
SOX2 FR	GCTTCTCCGTCTCCGACAAAA	
SALL2 F2	CAAGCCCCTACTACCCCTCT	
SALL2 R2	GAAAGGATGCTGTGACCCCA	
OLIG2 F2	CCAGAGCCCGATGACCTTTT	
OLIG2 R2	CACTGCCTCCTAGCTTGTCC	
PUO3F2 F2	CAGACCACCATCTGCAGGTT	
PUO3F2 R2	CTCGATGGAGGTCCGCTTTT	
a-ACTIN F	GTCAACCAGGAGAACGAGCA	
a-ACTIN R	GCAGCGTGTTGAAGTTGATC	
FN1 F	CCACACAGAACTATGATGCC	
FN1 R	ACGCTTGTGGAATGTGTCGT	
GAPDH F	GAGTCAACGGATTTGGTCGT	
GAPDH R	GATCTCGCTCCTGGAAGATG	
OLIG2-450 F	CACTTCCCACTCGTTTATTC	ChIP
OLIG2 + 50 R	AGGTCATCGGGCTCTGGC	
SALL2promF1	CACTGCCTCCTAGCTTGTCC	
SALL2promR1	CATTTGTAAGTCCAGGCCT	
SOX2promF2	CTCAGAGTTGATACAGACCT	
SOX2promR2	CACCCAGAAAGTCCTACTCT	
PUO3F2promF2	TGATGTATGAGGACGTTAT	
PUO3F22promR2	ACCGTTCAATTATGTGG	

### Chromatin immunoprecipitation (ChIP)

ChIP was performed on cells transfected as described above. The cross-link between protein and DNA was performed using formaldehyde 0.75% for 10 min at room temperature. Glycine (135 mM) was added to medium for 5 min at room temperature to quench the formaldehyde and terminate the cross-linking reaction. After 3 washes in phosphate-buffered saline, cells were harvested by the scraper of the plate. After centrifuge, cells were resuspended in ChIP lysis buffer (50 mM HEPES–KOH pH7.5, 140 mM NaCl, 1 mM EDTA pH8, 1% Triton X-100, 0.1% sodium deoxycholate, 0.1% SDS, protease inhibitors). Isolated chromatin was sonicated to obtain fragments ranging from 200 to 1000 bp.

FOSL1 and cross-linked DNA were immunoprecipitated using the antibody FRA1 (D80B4) produced in rabbit (mAb #5281, Cell Signaling technologies). The antibody was conjugated to Dynabeads (Thermo Fisher Scientific, Waltham, Massachusetts, USA) and immunoprecipitation was performed according to the manufacturer’s instructions.

The de-crosslinking reaction was performed at 70 °C for 10 min and DNA was isolated with QIAmp DNA Mini Kit (Qiagen, Hilden, Germany). Targets were analyzed by PCR performed as described in^38^, and using primers as reported in Table 1.

### Pluripotent stem cell array

Total proteins were isolated from HEK293T transfected with empty vector or pCMV-FOSL1, using a lysis buffer containing TrisHCl (pH 7.4, 50 mM), NaCl (150 mM), Triton (1% v/v), ethylenediaminetetraacetic acid (EDTA, 20 mM), phenylmethylsulfonyl fluoride (PMSF, 2 mM), protease and phosphatase inhibitors (Pierce, Rockford, IL, USA), leupeptin (2 ug/ml), glycerol (10% v/v), and water. Total proteins were quantified using Bradford reagent and NanoDrop 2000/2000c Spectrophotometers (Thermo Fisher Scientific). 100 μg of total proteins were then assayed with the Human Pluripotent Stem Cell Antibody Array (R&D Systems, Minneapolis, MN, USA) according to the manufacturer instructions. The acquisition of the chemiluminiscent signal of the membrane of the array was performed using ChemiDoc MP Imager (Bio-Rad). The analysis was performed using Image Lab Software (Bio-Rad) using the volume tool analysis methods.

### Cell aggregation assay

HEK-293T cells were counted, transfected in a suspension of OptiMEM medium (50 μl) containing lipofectamine 3000 and P3000 (0.5 μl each) and plasmids (10 ng) and plated in 96 well plates at 10,000 cells per well of confluence. Plates were coated with a basal layer of 100% (50 μl) and a layer of 2% Matrigel Growth Factor Reduced (GFR) Basement Membrane Matrix without supplement. Cells were photographed every 2 days starting from 24 h after the transfection.

### Western blot

30 μg of total proteins, isolated from HEK293T transfected with empty vector or pCMV-FOSL1 as described before, were separated using SDS-PAGE and transferred onto polyvinyldene difluoride (PVDF) membrane. After 2 h of blocking using not-fat dry milk at 5%, proteins were detected with specific primary antibodies at dilution of 1:1000: FRA1 (D80B4) (Cell Signaling technologies), FN1 (Sigma-Aldrich), a-ACTIN (Santa-Cruz), GAPDH (Cell Signaling technologies). After the incubation of secondary antibodies, the bands were detected with chemiluminescent using Clarity Western ECL substrate (BIO-RAD) and a charge-coupled-device camera (Chemidoc, BIO-RAD).

### Statistical analysis

Results are reported as means ± standard deviation. Differences were assessed with an unpaired t-test, and p-values lower than 0.05 were considered statistically significant. All statistical analyses were performed using GraphPad Prism version 5.0 statistical software (GraphPad Software Inc., San Diego, CA, USA).

## Supplementary Information

Supplementary Figures.

## Data Availability

This study did not generate new unique reagents. The samples used in this study are available under request.
