# Supplementary material for: The role of FOSL1 in stem-like cell reprogramming processes
Source: Sci Rep. 2021 Jul 19;11:14677. doi: 10.1038/s41598-021-94072-0 (PMC8290037; doi:10.1038/s41598-021-94072-0)
Supplement: Supplementary file 1 — Supplementary Figures. [file 41598_2021_94072_MOESM1_ESM.pdf]

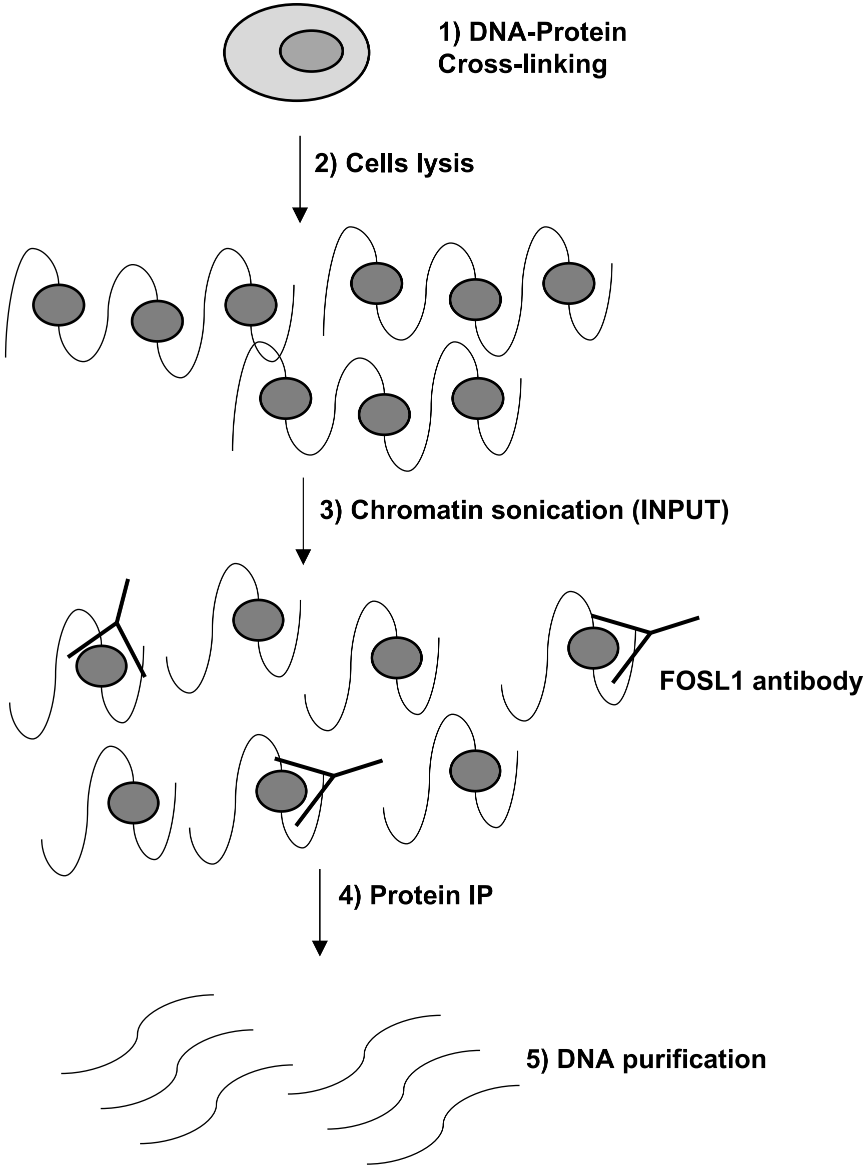

Supplementary figure 1. ChIP experiment. The steps of the ChIP experiment: 1) DNA-protein crosslinking; 2) cell lysis; 3) chromatin sonication; 4) protein immunoprecipitation using the antibody for FOSL1; and 5) DNA purification.

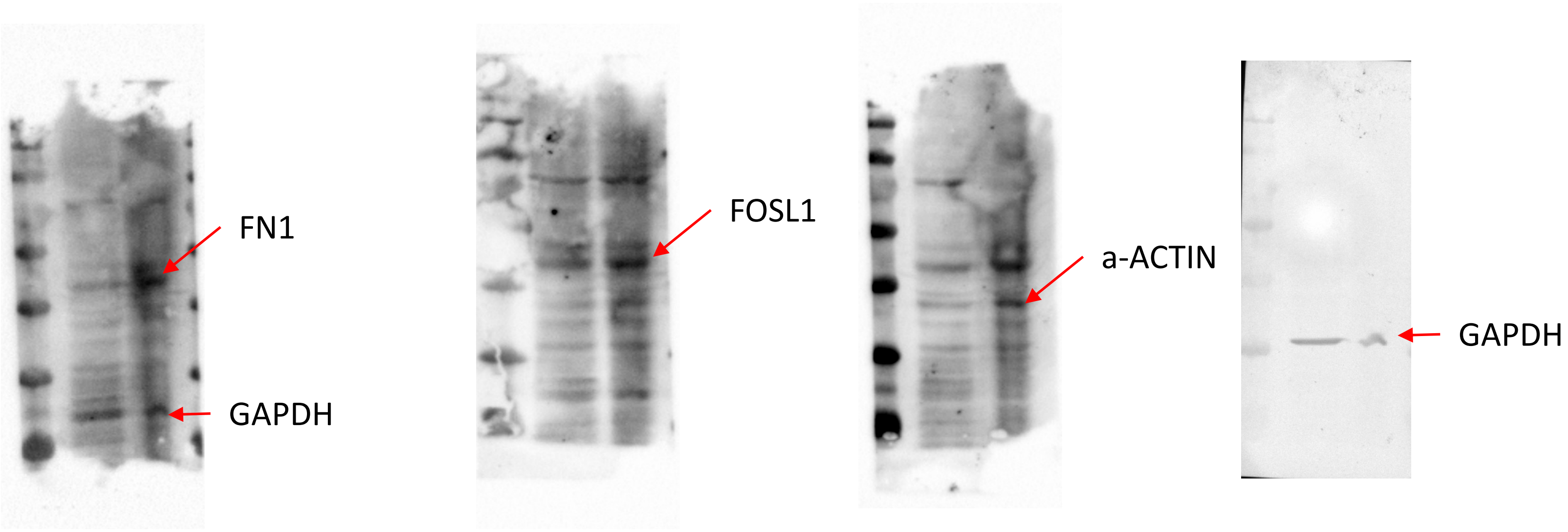

Supplementary figure 2. Entire membranes of western blot reported in Fig. 5D.
